# On the Use of Digitalis in Amenorrhœa, with Cases of Its Successful Administration

**Published:** 1832

**Authors:** W. T. S. Cornett

**Affiliations:** Versailles, Indiana


Art. II. *On the Use of Digitalis in Amenorrhœa,—Paramenia Obstructionis, with Cases of its successful Administration.* By W. T. S. Cornett, of Versailles, Indiana.

So far as I know, Digitalis has never as yet been noticed by the profession as possessing Emmenagogue properties, nor is it my purpose in this paper to contend that it exerts any direct or specific influence over the functions of the uterus, a remark which, I think, may with propriety be extended to the whole catalogue of Emmenagogue Medicines; but, that under certain circumstances Digitalis may be used with success in Amenorrhœa, and even where the ordinary remedies used in such cases, fail to restore the healthy functions of the uterus, the result of my experience leaves me in not the slightest shadow of doubt. The manner in which my attention was first directed towards this remedy in Amenorrhœa, was, I confess, accidental. I had conceived no views a priori of its modus operandi which were calculated to give origin to the belief that it might be used with advantage as an Emmenagogue. About five years since, Miss M—— U——, ætat. 19, came under my care, laboring under Paramenia Obstructions. There had been no appearance of the catamenia for the last twelve months. She was a girl of stout robust constitution and sanguine temperament. The case was produced by cold, from exposure to the night air. When she came under my care, the prominent symptoms were, pain in the heart, dry hacking cough, almost constant headache, constipation of the bowels, exacerbations of fever every afternoon, pulse small, tremulous, and slightly corded, loss of appetite, sallow complexion, and general anasarca. Also, pain in the epigastrium, distention of the stomach with flatus, acid eructations, &c. In the first place I adopted the usual course in such cases, which it is unnecessary to detail, with no alleviation whatever. The course was pursued until the case had advanced so far toward a fatal termination that I deemed it wholly unnecessary to continue the treatment. Her friends were, however, unwilling for me to discontinue my visits, and insisted on at least a palliative course. With a view of mitigating in some degree the distressing pectoral symptoms, I directed the Tinct. Digitalis, commencing with the usual dose, increasing the quantity daily until the system became sensibly under its influence. The result was, in a short time, relief of the difficulty of breathing, pain in the heart, &c., also, subsidence of the hydropic swellings, and a general improvement in appearance. The remedy was continued, with occasional intermissions, to prevent accumulation, for the term of six or eight weeks, when the menses appeared, bringing entire relief to all the symptoms. Within the following six months, one or two interruptions occurred to the regular appearance of the catamenia, but prompt relief was afforded by resuming the Digitalis. It will be proper to remark here, that from the commencement of the administration of the Digitalis, no other medicine was given except an occasional laxative. The result in this case induced me again to have recourse to our medicine in subsequent cases.

Case 2. In April, A. D. 1830, I was called to attend Miss J——, a hiredgirl, æt. 18, who had labored under Paramenia Obstructionis for the last six months, in consequence of having taken cold. When I first saw her she complained of pain in the heart, cough, headache, pain in the back, loins, and hips, evening exacerbations of fever, palpitation of the heart, loss of appetite, obstipatio intestinum, œdematous swellings of the feet and legs, and leucorrhœa. Previous to my having been employed in the case she had been treated by another physician without success. I determined to try the effects of the Digitalis, with the assistance of no other remedy except an occasional laxative, which was indispensable on account of constipation of the bowels. In nine days after commencing the Digitalis, the menses made a partial appearance, attended with considerable pain. The medicine was still continued with an occasional intermission, as in case I., and in four weeks, a copious flow of the catamenia, unattended with pain, ensued. Treatment was now discontinued, and my patient resumed her ordinary occupation, free from disease.

Case 3. In July, 1830, I was called to see Miss M——, who informed me that she had taken cold 18 months previous, which caused suppression of the menses. Medical aid was called in, and after a protracted treatment the menses made a partial appearance at irregular intervals, accompanied by severe pain in the back, loins, &c., constituting an aggravated case of Dysmenorrhœa. Her general health had gradually declined. When I was called to attend her she had again taken cold; the effect of which was entire suppression of the menses, accompanied with alarming hæmorrhage from the lungs; she also had pain in the heart, cough, and general emaciation. In the first place, measures were of course taken to arrest the hæmorrhage, after which, the Digitalis was used alone, as before. In two weeks, under its influence, the menses appeared, unattended with pain. The pulmonary symptoms immediately subsided, and a degree of health was restored, to which she had long been a stranger. I might enumerate other cases which would go equally well to establish the utility of Digitalis in Paramenia Obstructionis, but think it unnecessary. The above cases have been selected because other treatment had been previously adopted without success; and because of their long standing it cannot be reasonably inferred that the vis medicatrix naturæ alone produced the cures. It is not my design to enter the lists with those who cavil about the modus operandi of the Digitalis. I have given the the result of my experience with it in Paramenia Obstructionis, with the full belief that it is a remedy worthy the attention of the profession, in such cases as I have detailed, and hope its merits may be investigated by those who are more competent to judge than myself. It is well known to the profession, that medicines producing apparently the most opposite effects, do, occasionally, under different circumstances, produce a re-appearance of the catamenia. Suppression of the catamenia depends frequently on causes directly opposite, and, of course, the treatment must vary accordingly. Debility and lax fiber are frequent causes; but in my practice, which is chiefly in the country, it is depending on an opposite state of the system oftener than otherwise; and hence I infer, that Digitalis acts favorably by lessening the general excitement and producing that general relaxation of the system, and also of the vessels of the uterus which is necessary to the re-establishment of healthy action; and also, it may act favorably by determining to the kidneys. Notwithstanding all the means that have hitherto been employed by the profession, there are yet many cases of Amenorrhœa that wear on until death winds up the catastrophe. Whether this consequence is sometimes inevitable, or whether it is owing to the yet imperfect state of pathological views and remedial means, I am not prepared to say. But certain I am, that there is no class of affections that call more loudly upon the energies of the profession. There is no disease to which the female sex is liable, that is regarded with more anxiety, and perhaps none more to be dreaded in its progress toward laying waste the constitution, than disturbed uterine function. Upon the healthy performance of this law of her nature the brightest hopes of the female are suspended. Destroy this, and you destroy, (I had almost said,) the whole object of her life. Her day dreams of happiness had been transient as the mists of man: she looks abroad upon society with the feelings of one never destined to enjoy its blessings. The whole face of nature has become desolate and dreary as the shades of night, or the barren wilds of an unfrequented desert;—friends and society have lost their charms, and the landscape its beauty. The azure vault of heaven, bespangled with its myriads of luminaries, cheers not her fading soul;—the clouds that hang on high, are the mourning curtains of the universe, and the rolling of the distant thunder is but the knell of all her hopes, save that of Heaven.

Editorial Note. Since reading the above article we have met with the following remarks upon the use of digitalis in chlorosis, which without at all detracting from the merit of the author of this essay, go to prove that the tincture of digitalis has been successfully resorted to by other physicians.

"When effusion has taken place into the cavities of the body, occasioning difficult breathing, attended by œdema and a scanty secretion of urine, diuretics are the most efficacious remedies; and of these, the most reliance may be placed on digitalis, of which, ten drops of the tincture, with twenty of nitrous ether may be given three times a day. This medicine, by promoting the secretion of urine, relieves the breathing and other symptoms so effectually that Dr. Hamilton, who was much struck with its effects, recommends it as the most effectual remedy in all the stages of chlorosis; supposing this disease to depend upon a weakened condition of the lymphatic system, which is stimulated by this combination into a healthy activity. Dr. Hamilton prescribes one part of the tinct. of digitalis with two parts of nitrous ether, of which mixture thirty drops may be given every hour, until a copious secretion of urine is produced. This evacuation being once excited to a considerable amount, he then keeps up an action, both on the kidneys and bowels, by administering such doses of tartar emetic as may be borne without sickness, every six hours. After continuing this treatment for three weeks, he then recommends the tonic remedies, such as steel, the aloetic and stomachic bitters, &c. If vertigo or syncope should occur during the use of digitalis, Dr. Hamilton states that it is effectually relieved by the use of the warm bath."—*Gooch's Compendium of Midwifery.*

